# An investigation of the neural circuits underlying reaching and reach-to-grasp movements: from planning to execution

**DOI:** 10.3389/fnhum.2014.00676

**Published:** 2014-09-02

**Authors:** Chiara Begliomini, Teresa De Sanctis, Mattia Marangon, Vincenza Tarantino, Luisa Sartori, Diego Miotto, Raffaella Motta, Roberto Stramare, Umberto Castiello

**Affiliations:** ^1^Department of General Psychology and Center for Cognitive Neuroscience, University of PadovaPadova, Italy; ^2^Department of Medicine, University of PadovaPadova, Italy

**Keywords:** reach-to-grasp, reaching, functional magnetic resonance imaging, motor planning, motor execution

## Abstract

Experimental evidence suggests the existence of a sophisticated brain circuit specifically dedicated to reach-to-grasp planning and execution, both in human and non-human primates (Castiello, 2005). Studies accomplished by means of neuroimaging techniques suggest the hypothesis of a dichotomy between a “reach-to-grasp” circuit, involving the anterior intraparietal area, the dorsal and ventral premotor cortices (PMd and PMv – Castiello and Begliomini, 2008; Filimon, 2010) and a “reaching” circuit involving the medial intraparietal area and the superior parieto-occipital cortex (Culham et al., 2006). However, the time course characterizing the involvement of these regions during the planning and execution of these two types of movements has yet to be delineated. A functional magnetic resonance imaging study has been conducted, including reach-to-grasp and reaching only movements, performed toward either a small or a large stimulus, and Finite Impulse Response model (Henson, 2003) was adopted to monitor activation patterns from stimulus onset for a time window of 10 s duration. Data analysis focused on brain regions belonging either to the reaching or to the grasping network, as suggested by Castiello and Begliomini (2008). Results suggest that reaching and grasping movements planning and execution might share a common brain network, providing further confirmation to the idea that the neural underpinnings of reaching and grasping may overlap in both spatial and temporal terms (Verhagen et al., 2013). But, although responsive for both actions, they show a significant predominance for either one of the two actions and such a preference is evident on a temporal scale.

## INTRODUCTION

The reach-to-grasp movement has been investigated from many perspectives and through different approaches given that it represents an ideal experimental window to elucidate action–perception interactions. Studies centered on motion analysis of grasping have shown that the final posture of hand and fingers in contact with the object represents the end result of a motor sequence starting well ahead of the action of grasping itself (Jeannerod, 1984; Gentilucci et al., 1991; Jakobson et al., 1991; Chieffi and Gentilucci, 1993). The progressive shaping of hand and fingers is accomplished through a progressive opening of the grip with straightening of the fingers, followed by a closure of the grip until the size of the object is perfectly matched. The point in time where grip size is the largest (maximum grip size) is a clearly identifiable landmark that occurs well before the fingers come into contact with the object (Jeannerod, 1984). Many studies have showed that even very subtle changes in object properties can result in a significant change in grasping kinematic parameters (see Smeets and Brenner, 1999, for a review). The susceptibility of kinematic parameters demonstrates how sensitive and sophisticated are the processes responsible for the “translation” of object properties into the motor program implemented during the “hand preshaping” stage are.

In neural terms, neurophysiological studies in non-human primates have demonstrated that reaching and grasping movements, even if embedded in the same act, are coded by different neural systems. Computations regarding the grasp component seem to occur within a lateral parietofrontal circuit involving mainly the anterior intraparietal area (AIP) and both the dorsal (PMd) and the ventral (PMv) regions of premotor areas (Moll and Kuypers, 1977; Godschalk, 1991; Raos et al., 2004). Computations regarding the reaching component, instead, seem to occur within a more medial parieto-frontal circuit including the medial intraparietal area (mIP) at the boundaries with area V6A (Andersen and Cui, 2009), and the PMd (Sakata and Taira, 1994).

Neuroimaging and transcranial magnetic stimulation (TMS) studies in humans go in the same direction (for review see Castiello, 2005; Culham et al., 2006; Olivier et al., 2007; Begliomini et al., 2008). Several studies agreed on the key role played by the human AIP (hAIP) in grasping behavior (Grafton et al., 1996; Dohle et al., 2000; Culham et al., 2003, 2006; Frey and Gerry, 2006; Begliomini et al., 2007a, 2008; Filimon, 2010) and it has also been proposed the inferior frontal gyrus (IFG) and the dorsal part of the middle frontal gyrus (MFG) at the boundaries with the precentral gyrus (PreCG) as the human homologs of monkey F2 and F5 (Davare et al., 2006; Begliomini et al., 2007b, 2008). Rather, a pathway including the superior part of the parieto-occipital cortex (SPOC), the medial intraparietal area (mIP) and the PMd has been suggested as the neural substrate for planning and execution of reaching movements (Connolly et al., 2003; Prado et al., 2005; Culham et al., 2006; Cavina-Pratesi et al., 2010; Filimon, 2010; Vesia and Crawford, 2012).

The dichotomy between a lateral fronto-parietal network supporting grip formation and a medial fronto-parietal network being the neural underpinning of reaching has recently been put into question. Evidence from single-cell data (Raos et al., 2004; Fattori et al., 2009, 2010) and lesion studies (Battaglini et al., 2002) suggests that the parieto occipital area V6a and dorsal premotor area F2 are also involved in managing specific aspects of grasping behavior such as grip posture and wrist orientation. For example, reaching-related neurons in macaque area V6A appear to be sensitive not only to reach direction (Fattori et al., 2004), but also to target orientation (Galletti et al., 1999; Fattori et al., 2009), target shape (Fattori et al., 2012), and grasp configuration (Fattori et al., 2010). Similarly, functional magnetic resonance imaging (fMRI) investigations in humans reported grasping-related parieto-occipital and dorsal premotor cortex activations (Chapman et al., 2002; Begliomini et al., 2007a,b, 2008; Gallivan et al., 2011), which might be considered the possible human homolog for monkey areas V6A and F2, respectively. Moreover, a recent neuroimaging study, based on the effective parieto-frontal connectivity, argues against the existence of dedicated circuits for reaching and grasping (Grol et al., 2007). The results of this study show that while grasping large objects increases connectivity among areas belonging to the dorso-medial circuit, grasping small objects increases inter-regional couplings mainly within the dorsolateral circuit: however, a certain degree of overlap between the two circuits was observed. Along the same line, a recent multi-voxel pattern analysis (MVPA) study provides further evidence against a segregation of reaching and grasping circuits, showing that both grip types and reach direction are coded within the inferior portion of the dorsal premotor cortex (iPMd), PMv, AIP, primary motor (M1), somatosensory (S1) cortices, and the anterior superior parietal lobe (SPLa – Fabbri et al., 2014).

Overall, these findings indicate that in humans, like in monkeys, reach-to-grasp movements involve a large network of interconnected structures in the parietal and frontal lobes (Rizzolatti and Luppino, 2001; Brochier and Umiltà, 2007; Castiello and Begliomini, 2008). However, how these areas interact has yet to be fully clarified. The majority of studies has focused on the question of “if” or “how” these areas interact during grasping or reaching execution, neglecting the possibility that interaction patterns could change across time, according to action stages (Verhagen et al., 2013).

In this respect, the functional distinction between the pre-movement planning and the control stages of action has been the subject of much investigation (e.g., Woodworth, 1899; Vince, 1948; Fitts, 1954; Keele, 1968; Beggs and Howarth, 1970, 1972; Carlton, 1981; Meyer et al., 1988; see Glover, 2004 for a review). And the existence of these two stages has generally become accepted as an underlying principle of human motor behavior (Jeannerod, 1988; Rosenbaum et al., 1990; Rosenbaum, 1991).

In neural terms, the functional distinction between planning and execution has been investigated in a variety of studies (e.g., Grol et al., 2007; Bozzacchi et al., 2012; Glover et al., 2012). Grol and colleagues used Dynamic Causal Modeling (Friston et al., 2003) on fMRI timeseries acquired during planning and execution of visually guided reaching-to-grasp movements toward objects of different size to explore the interregional couplings between regions of the dorsolateral (AIP and PMv) and the dorsomedial (V6A and PMd) circuits. By assessing how different hand–object interactions modulated the effective connectivity within these networks, they demonstrated that the involvement of the dorsolateral and dorsomedial parieto-frontal circuits is largely related to the degree of online control required by the prehension movement (Grol et al., 2007).

Another study provides an attempt to contrast activity related to planning and online control in the human brain during simple reaching and grasping movements (Glover et al., 2012). These findings provide evidence that the planning and control of even simple reaching and grasping actions use different brain regions, including different parts of the frontal and parietal lobes. Movement planning determined activity within the superior temporal sulcus (STS), the pre-supplementary motor area (pre-SMA), the mIP, the SPOC, the PM, and the insula. Movement execution, instead, seems to be supported mainly by the sensorimotor cortex, the cerebellum, the SMA, the supramarginal gyrus (SMG), and the superior parietal lobe (SPL).

Pre-movement cortical activity related to reaching and grasping tasks has also been studied by means of motor-related cortical potentials (Bozzacchi et al., 2012). In this study, different activity patterns in terms of onset, amplitude, duration, and sources were recorded in the preparation phase according to the specific action. The results indicate the presence of parietal activity, well before the action begins, for goal-oriented actions such as grasping an object but not in reaching. This activity starts about two seconds prior to the action and is maximal about one second later in the areas contralateral to the used hand. Moreover, the type of action to be performed also modulates motor preparation in terms of timing and intensity of the different brain activity.

Along these lines, we hypothesized that (i) action planning might be characterized by a prominent contribution of decision-related areas, in charge of choosing the grasping schema to be implemented according to object properties, position, and action goals. Differently, action execution might be characterized by a larger contribution of motor-related areas. In addition (ii) we aimed to disentangle interactions between dorsolateral and dorsomedial circuits not only during the actual execution of reaching and grasping movements, but also during their planning. Finally (iii) concerning grasping, we hypothesized that different grasping schemata (e.g., precision grip and whole hand grasp) could be characterized by different neural underpinnings during both movement planning and execution.

To test these hypotheses, we instructed participants to perform reaching or grasping movements, toward a small or a large spherical object, while lying in a magnetic resonance (MR) scanner. Action stages (planning and execution) were distinguished and segregated by acoustic cues presented through headphones. To monitor temporal dynamics of interaction patterns within the fronto-parietal network a Finite Impulse Response (FIR – Henson, 2003) model was adopted for BOLD signal modeling. Importantly, with respect to previous studies we subdivided the time course of activation to determine brain activity related to the pre-movement planning and online control of reaching and grasping in humans. Prior to movement initiation, planning is entirely responsible for the initial determination of all movement parameters, and continues to be highly influential early in the movement. As movements progress, however, the influence of control on the spatial parameters of the action increases. Can such a gradual crossover between planning and control systems being evident through the temporal unfolding of neural activity?

## MATERIALS AND METHODS

### PARTICIPANTS

Eighteen volunteers (six men, 12 women, range 20–31 years old) participated in the study. All participants fulfilled the inclusion criteria suggested by the Italian Society of Medical Radiology, none had a history of neurological, major medical, or psychiatric disorders. They were all right-handed according to the Edinburgh Handedness Inventory (Oldfield, 1971). Experimental procedures and scanning protocols were approved by the University of Padua Ethics Committee and conducted in accordance with the Declaration of Helsinki (Sixth revision, 2008). All participants gave their informed written consent to participate in the study.

### TASK AND STIMULI

**FIGURE 1:**
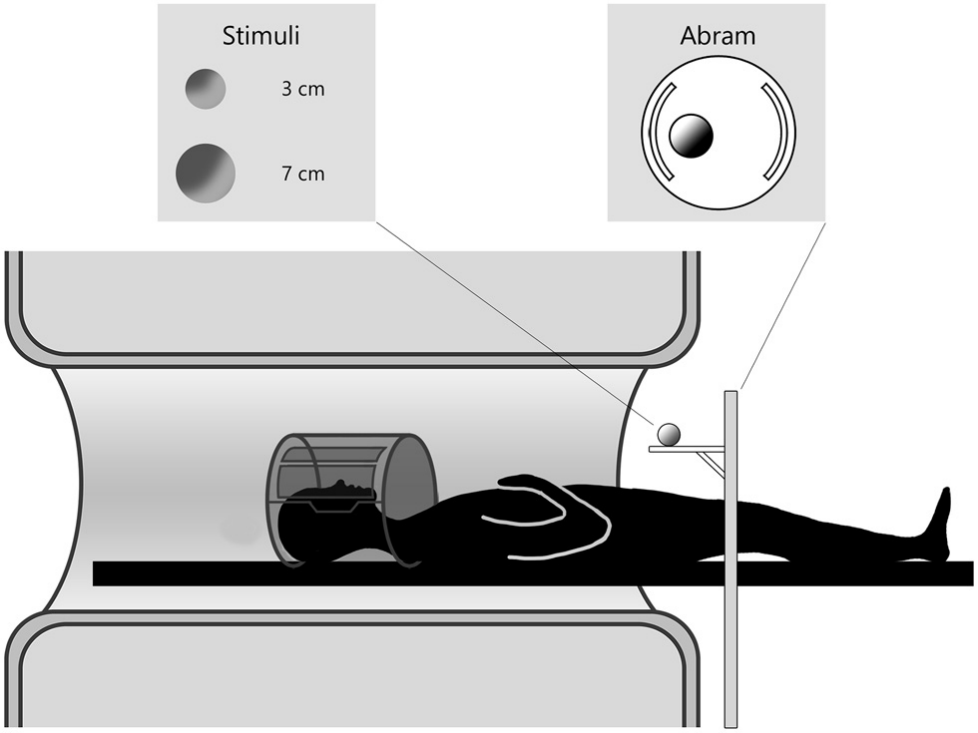
**Experimental setup of data acquisition.** The participant is laying in the MR scanner and the motorized platform ABRAM is presenting stimuli following a sequence administered by a PC in the control room. The position of the rotating platform plus a pillow slightly tilting the head allow for direct viewing of the stimuli.

Three dimensional (3D) stimuli were presented by means of an MR compatible motorized circular rotating table (ABRAM^1^; **Figure 1**). The experimental stimuli consisted of two wooden spheres of different dimensions (a small wooden sphere of 3 cm diameter and a large wooden sphere of 7 cm). Participants were requested to perform two different kinds of movement: (i) reach toward and grasp the stimulus; (ii) reach the stimulus with the hand in a fist posture. All participants naturally adopted a precision grip, the opposition between the index finger and thumb to grasp the small stimulus, and whole hand prehension in which all fingers were opposed to the thumb to grasp the large stimulus. During movement execution, participants were requested to keep the eyes on the stimulus. To facilitate direct viewing of the stimulus the head was tilted (10–15°) by means of foam MRI compatible cushions. Given that participants performed the actions with the right hand, a further cushion was placed under the upper right arm, in order to minimize discomfort during the movement. Trials structure was the following: (i) an acoustic cue delivered through MR-compatible headphones indicated the type of movement to perform. A single tone indicated a reach to grasp movement (duration 300 ms; frequency 1600 Hz); a double pulse tone indicated a reaching only movement (each pulse lasted 70 ms with a frequency of 400 Hz). The interval between the two pulses was of 60 ms and the total duration of the tone was 200 ms; (ii) the acoustic cue was followed by a 2 s delay; and (iii) a “go” signal was presented (a whistle of 200 ms duration; frequency 440 Hz). Participants were requested to wait for the “go” signal to begin the movement indicated by the acoustic cue. Participants were trained to familiarize with the acoustic instructions during a training session before scanning. They were requested to perform the movement at a natural speed.

### EXPERIMENTAL DESIGN

The entire experiment consisted of four runs of 45 trials each, in which stimulus size (small, large) was randomized across runs and type of movement (grasping, reaching) was randomized within runs. Therefore the design (factorial 2 × 2) included four experimental conditions: reach to grasp toward a small stimulus (GS), reach to grasp toward a large stimulus (GL), reaching only toward a small stimulus (RS), reaching only toward a large stimulus (RL). Since stimulus size was randomized across runs, for each run two movements had to be performed, either grasping or reaching. A mixed design was adopted, grouping trials belonging to the same type (grasping or reaching) in short sequences of different numerosity (varying from 3 to 5 trials of the same type). This approach has been adopted on one hand in order to control for predictability phenomena, possibly induced by trials sequences of constant length. On the other hand, continuous changes in task request (e.g., RS-GS-RS-GS-RS and so on) could have resulted in task-switching related activity. Variable interstimulus interval (ISI) was considered, including durations from 3 to 6 s according to a long exponential probability distribution (Dale, 1999; Hagberg et al., 2001). ISI duration was independently randomized within each single experimental run.

### DATA ACQUISITION

The experiment was carried out on a whole body 1.5 T scanner (Siemens Avanto) equipped with a standard Siemens eight channels coil. Functional images were acquired with a gradient-echo, echo-planar (EPI) T2^∗^-weighted sequence in order to measure blood oxygenation level-dependent (BOLD) contrast throughout the whole brain (37 contiguous axial slices acquired with descending interleaved sequence, 56 × 64 voxels, 3.5 mm × 3.5 mm × 4.0 mm resolution, FOV = 196 mm × 224 mm, flip angle = 90°, TE = 49 ms). Volumes were acquired continuously for each run with a repetition time (TR) of 3 s; 102 volumes were collected in each single scanning run, resulting in functional runs of 5 min and 25 s duration (21 min and 40 s of acquisition time in all). High-resolution T1-weighted image were acquired for each subject (3D MP-RAGE, 176 axial slices, no interslice gap, data matrix 256 × 256, 1 mm isotropic voxels, TR = 1900 ms, TE = 2.91 ms, flip angle=15°).

### DATA ANALYSIS

Data preprocessing and analysis were performed using SPM8 (Statistical Parametric Mapping, Wellcome Institute of Cognitive Neurology, London, UK) implemented in MATLAB 7.5.0 environment (MathWorks, Natick, MA, USA). For each participant, the first two volumes of each fMRI run were discarded because of the non-equilibrium state of the magnetization in order to allow for stabilization. ArtRepair toolbox (ArtRepair software Package, for SPM^2^) was adopted in order to correct for possible images corruption due to signal spikes induced by head motion. Motion correction was carried out by realigning and unwarping data. Structural images were segmented and subsequently the image of gray matter was co-registered with all the functional images. Structural and functional images were then normalized adopting the template provided by the Montréal Neurological Institute (MNI) implemented in SPM8. Finally, functional images were spatially smoothed using a 7 mm × 7 mm × 8 mm full-width-at half-maximum (FWHM) Gaussian Kernel. At the end Artrepair toolbox was applied in order to identify and correct large scan-to-scan head motion, which may result in large global intensity changes. First-level analysis was carried out by adopting an FIR (Henson, 2003), in order to characterize the temporal evolution of the hemodynamic response (HR) without *a priori* hypothesis on its shape The peculiarity of the FIR model is the absence of assumptions about the shape of the HR: this feature allows for the splitting of a selected post-stimulus time window into different temporal segments (a number of successive Time Bins (TB) by providing a set of basis functions within the framework of a general linear model (GLM). These basis functions can be considered as separate parameters (Dale and Buckner, 1997) and can be entered into the GLM model with time as a factor (Henson, 2003). According to this model, task-related BOLD variations were monitored from stimulus onset (cue signal), in order to capture BOLD variations related to both action planning (cue) and execution (go). In this respect, a simple canonical HRF model would have been not appropriate to capture signal variations related to all action stages: the structure of the trial includes different action stages lasting for a prolonged time (cue-go interval of 2 s plus action occurring thereafter). From this perspective the FIR model provides a more sensitive and detailed signal modeling, allowing for a monitoring of BOLD variations related to all trial stages. A post-stimulus time window of 10 s length was considered, starting from cue onset, and divided into 10 TB of 1 s each. TB width was lower than the TR used during data acquisition (3 s) because we attempted to specifically characterize differences at the subsequent stages of action planning and execution. In addition, it has also been shown that it is possible to sample the impulse response at post-stimulus intervals shorter than TR by jittering event onsets with respect to scan onsets (Josephs et al., 1997; Schilbach et al., 2008). In our study interstimulus interval varied from 3 to 6 s and had a jittered distribution. Reaching (RS and RL) and grasping (GS and GL) movements were modeled as separate events for each participant. Errors in action execution or missed trials were modeled as separate regressors of no interest. T-contrasts were computed for each condition (RS, RL, GS, and GL), in order to capture condition-specific HR variations for each condition in single TB (10 images per condition in all). Image analyzes were carried out after high-pass filtering (154 s) to remove subject-specific, low-frequency signal drifts and global intensity scaling. Following the estimation of a GLM for each single participant, effects for each experimental condition were tested by applying appropriate linear contrasts to the parameter estimates, resulting in a *t*-statistic for each voxel (SPMt). Images for each experimental regressor/condition were entered in a second level random effect analysis (RFX) allowing for inference to the general population, with type of movement (reaching, grasping) and stimulus dimension (small, large) as factors across the considered TBs (2 × 2 × 10; 40 images in all for each participant). With the purpose of clearly localize the neural substrates underlining the proposed reach to grasp or reaching only tasks, the analysis was conducted by adopting a searching mask built by several regions of interest, on the basis of available literature (for review see Castiello and Begliomini, 2008), suggesting a primary distinction between planning and execution-related areas. According to this distinction, the dorsolateral region of the prefrontal cortex (Rizzolatti and Luppino, 2001) and the anterior cingulate area (Matelli et al., 1991) would be mainly involved in movement planning, while the primary motor (Glover et al., 2005; Tunik et al., 2005; Rice et al., 2006) and premotor cortices (Culham et al., 2003; Frey et al., 2005; Begliomini et al., 2007b), as well as the parietal cortex (Binkofski et al., 1998; Culham et al., 2006; Begliomini et al., 2007a) would play a substantial role in action execution. In addition, also the SPL was included, as a brain region known to be involved in reaching control (Culham et al., 2003). The toolbox WFU PickAtlas (Wake Forest University^3^) was adopted to build the mask involving all the mentioned areas.

## RESULTS

### GLOBAL ANOVA

The interaction between type of movement, stimulus dimension and TBs was significant for several portions of the considered mask (see **Table 1**). Results are 0.001 uncorrected for multiple comparisons (*k* ≥ 20). This analysis underlined that the PreCG (Brodmann Area, BA 6) in the right hemisphere, and the inferior parietal lobule (IPL, BA 40) together with the anterior cingulate cortex (aCC) exhibited significant effects.

**Table 1:** Brain regions showing interaction effects between type of movement (grasping, reaching) and stimulus dimension (small, large) across all 10 Time Bins.

Region	BA	Hemisphere	*k*	MNI coordinates			*F*	*p*
				*x*	*y*	*z*		
**Precentral gyrus**	**6**	**Right**	**33**	**31**	**–14**	**70**	**3.63**	**0.000**
**Inferior parietal lobule (pIPS)**	**40**	**Left**	**29**	**–47**	**–60**	**46**	**3.75**	**0.000**
Inferior parietal lobule (aIPS)	40	Left		–47	–49	46	3.40	0.000
**Anterior cingulate gyrus**	**32**	**Left**	**21**	**–1**	**35**	**14**	**3.60**	**0.000**
Anterior cingulate gyrus	32	Left		–1	39	26	3.40	0.000

Results are obtained by means of a RFX analysis. Coordinates refer to the Montréal Neurological Institute (MNI) stereotaxic space. *p* values are uncorrected for multiple comparisons (0.001, *k* ≥ 20). BA, Brodmann area.

To better characterize our results, and in order to elucidate the possible evolution of interaction patterns across the whole post-stimulus window (10 s), separate ANOVA were conducted for each TB, considering type of movement (grasping; reaching) and stimulus dimension (small; large) as factors.

### SINGLE BIN ANOVA

Statistical threshold was set to *p* < 0.001, uncorrected for multiple comparisons and the adopted cluster extension was set to *k* ≥ 12.

### TB 1–3

Random effect analysis performed on TB 1, 2, and 3 did not bring to any significant result – neither main nor interaction effects.

### TB 4

**FIGURE 2:**
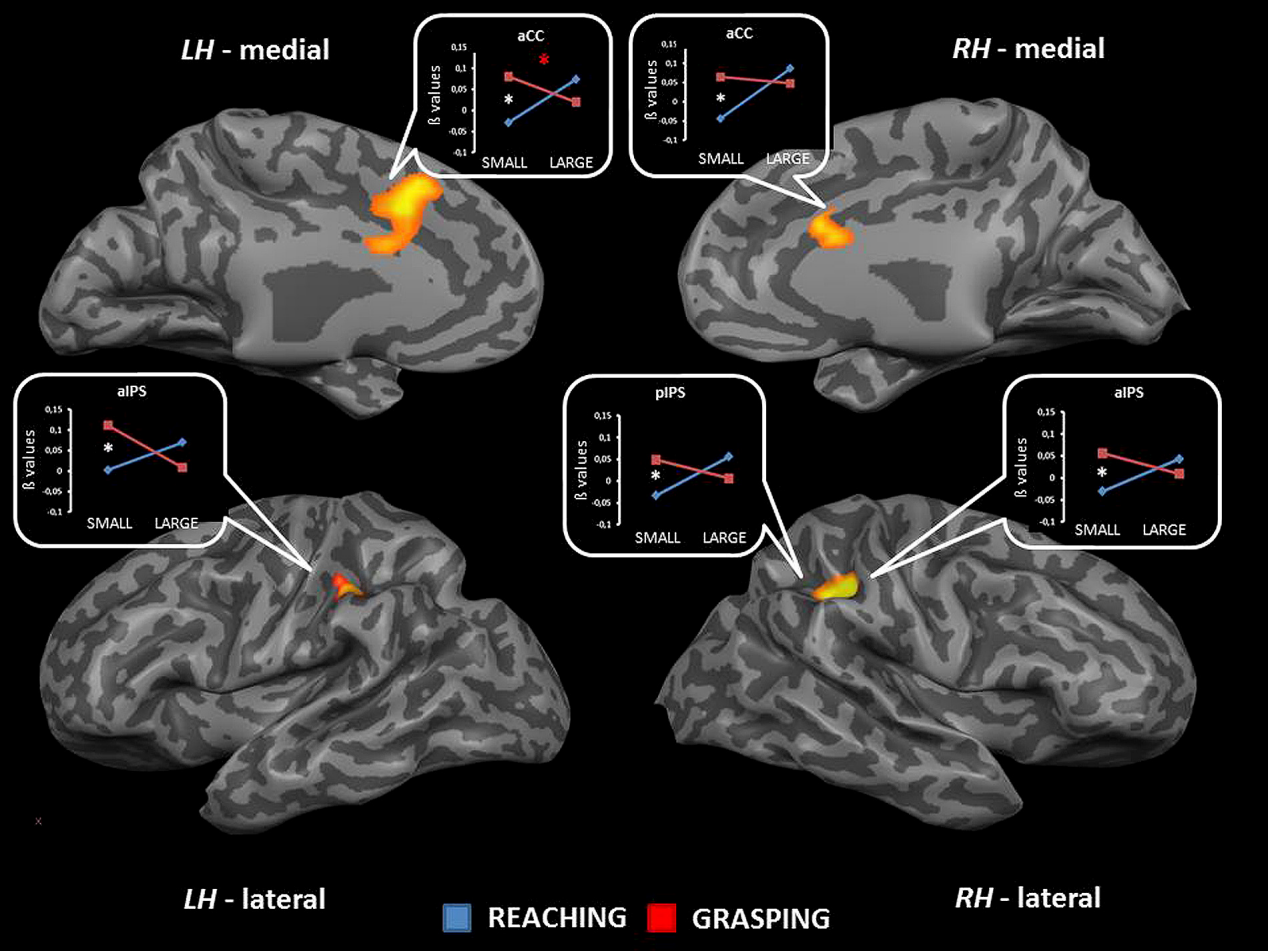
**Brain regions showing significant interaction effects between type of movement and stimulus dimension in TB 4.** The *p* value is set to 0.001, uncorrected for multiple comparisons, cluster size *k* ≥ 13. White asterisks indicate significant effects for the contrast GS > RS; red asterisks indicate significant effects for the contrast GS > RS. (aCC, anterior cingulate cortex; aIPS, anterior intraparietal sulcus; pIPS, posterior intraparietal sulcus; LH, left hemisphere; RH: right hemisphere; medial, medial view; lateral, lateral view).

The interaction between type of movement and stimulus dimension was significant for the IPL bilaterally (BA 40), within both anterior and posterior sector of the right intraparietal sulcus [aIPS: *F*_(1,68)_ = 21.36, and pIPS: *F*_(1,68)_ = 15.72, respectively], and the left aIPS [*F*_(1,68)_ = 21.52] and the aCC bilaterally [BA 32; left side: *F*_(1,68)_ = 19.92; right side: *F*_(1,68)_ = 16.09]. A close inspection of the interaction effects revealed a similar pattern of results for all the considered regions, that is RL determined a higher level of activation than RS; vice versa, GS seems to be associated with a higher level of activity than GL. *Post hoc* contrasts revealed that only the comparisons GS > RS and GS > GL were significant. In detail, the contrast GS > GL became significant only within the left aIPS, whereas the comparison GS > RS reached significance in all areas showing interaction effects (see **Table 2** and **Figure 2**). No further significant effects were observed.

**Table 2:** Brain regions showing interaction effects between type of movement (grasping, reaching) and stimulus dimension (small, large) distinguished in single Time Bins.

Region	BA	Hemisphere	*k*	MNI coordinates			*F*	*p*
				*x*	*y*	*z*	

**TIME BIN 1**

**TIME BIN 2**				**N.S.**			

**TIME BIN 3**				**N.S.**			

**TIME BIN 4**				**N.S.**			
**Inferior parietal lobule (aIPS)**	**40**	**Left**	**13**	**–43**	**–49**	**50**	**21.52**	**0.000**
**Inferior parietal lobule (pIPS)**	**40**	**Right**	**28**	**41**	**–56**	**50**	**21.36**	**0.000**
Inferior parietal lobule **(aIPS)**	40	Right		45	–42	50	15.72	0.000
**Anterior cingulate gyrus**	**32**	**Left**	**45**	**–1**	**18**	**46**	**19.92**	**0.000**
Anterior cingulate gyrus	32	Right		3	21	34	16.09	0.000
**TIME BIN 5**								

**Middle frontal gyrus**	**6**	**Right**	**15**	**48**	**0**	**42**	**20.72**	**0.000**
**Anterior cingulate gyrus**	**32**	**Right**	**20**	**–1**	**7**	**46**	**18.57**	**0.000**
Anterior cingulate gyrus	32	Left		10	11	42	17.70	0.00
**TIME BIN 6**								

**Middle cingulate gyrus**	**24**	**Left**	**26**	**–1**	**–4**	**50**	**20.68**	**0.000**
**Inferior parietal lobule (pIPS)**	**40**	**Right**	**14**	**38**	**–53**	**50**	**19.84**	**0.000**
**Inferior parietal lobule (aIPS)**	**40**	**Left**	**17**	**–40**	**–46**	**54**	**16.31**	**0.000**
**TIME BIN 7**								

**Precentral gyrus**	**4**	**Left**	**76**	**–40**	**–21**	**62**	**22.98**	**0.000**
Precentral gyrus	6	Left		–26	–14	74	19.30	0.000
Middle frontal gyrus	6	Left		–29	–11	66	18.78	0.000
**TIME BIN 8**								

**Inferior parietal lobule**	**40**	**Left**	**112**	**–61**	**–35**	**42**	**31.13**	**0.000**
Inferior parietal lobule (aIPS)		Left		–43	–46	50	28.91	0.000
Inferior parietal lobule (pIPS)		Left		–29	–67	38	23.80	0.000
**TIME BIN 9**								

**TIME BIN 10**				**N.S.**				

				**N.S.**				

Results are obtained by means of a RFX analysis. Coordinates refer to the Montréal Neurological Institute (MNI) stereotaxic space. *p* values are uncorrected for multiple comparisons (0.001, *k* ≥ 13). BA, Brodmann area.

### TB 5

**FIGURE 3:**
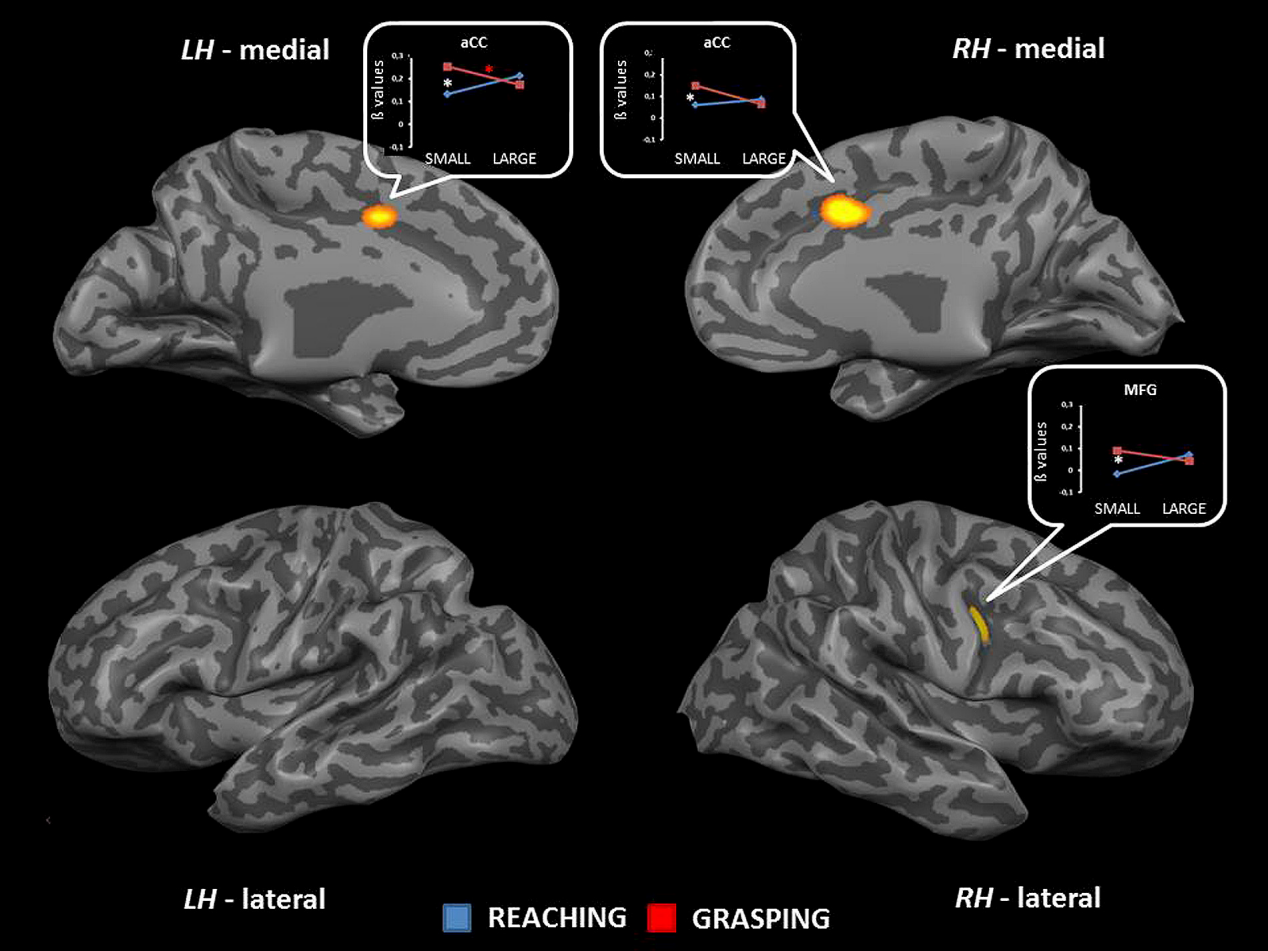
**Brain regions showing significant interaction effects between type of movement and stimulus dimension in TB 5.** The *p* value is set to 0.001, uncorrected for multiple comparisons, cluster size *k* ≥ 13. White asterisks indicate significant effects for the contrast GS > RS; red asterisks indicate significant effects for the contrast GS > RS. (aCC, anterior cingulate cortex; MFG, middle frontal gyrus; LH, left hemisphere; RH, right hemisphere; medial, medial view; lateral, lateral view).

The interaction between type of movement and stimulus dimension was significant for the right MFG [MFG, BA 6; *F*_(1,68)_ = 20.72] and the aCC (BA 32) bilaterally [right: *F*_(1,68)_ = 18.57; left: *F*_(1,68)_ = 17.70]. In both circumstances RL determined a higher level of activity than RS. Conversely, activity for GS was higher than that for GL. *Post hoc* comparisons revealed that only the contrast GS > RS and GL > GS reached significance. The contrast GS > RS led to significant differences in both the MFG and the aCC, both in the left and the right hemisphere. The contrast GL > GS showed significant effects only within the left aCC (see **Table 2** and **Figure 3**). No further significant effects were observed.

### TB 6

**FIGURE 4:**
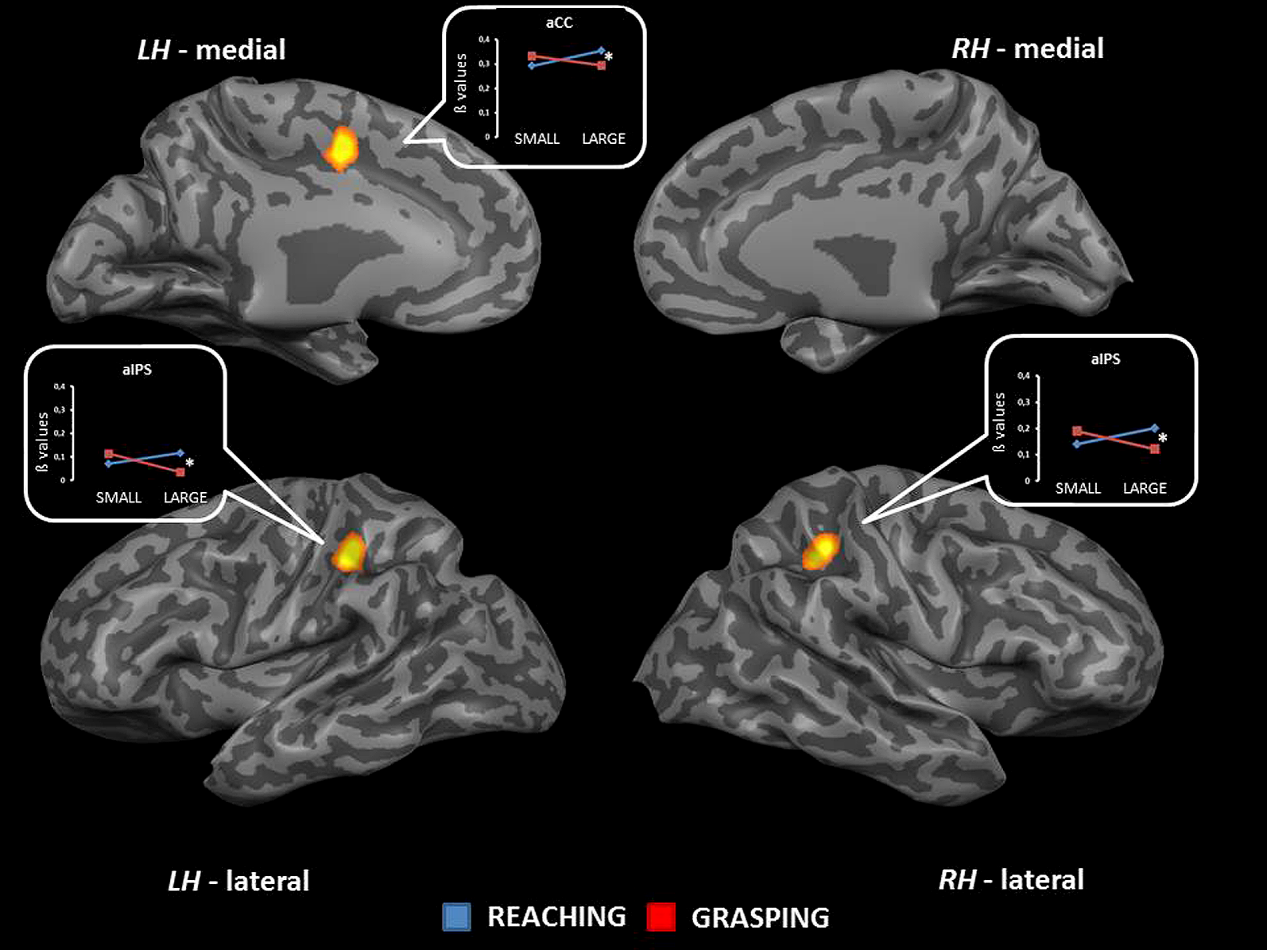
**Brain regions showing significant interaction effects between type of movement and stimulus dimension in TB 6.** The *p* value is set to 0.001, uncorrected for multiple comparisons, cluster size *k* ≥ 13. White asterisks indicate significant effects for the contrast GS > RS. (aCC, anterior cingulate cortex; aIPS, anterior intraparietal sulcus; LH, left hemisphere; RH, right hemisphere; medial, medial view; lateral, lateral view).

The interaction between type of movement and stimulus dimension was significant for the left aIPS [BA 40; *F*_(1,68)_ = 16.31], the right pIPS [BA 40; *F*_(1,68)_ = 19.84], and within the left middle cingulate cortex [mCC, BA 24; *F*_(1,68)_ = 20.68]. For these regions, the level of activity was higher for RL than for RS. Conversely, GS was associated with a level of activity higher than that observed for GL. More in detail the difference between RL and GL became significant in all regions showing interaction effects (see **Table 2** and **Figure 4**). No further significant effects were observed.

### TB 7

**FIGURE 5:**
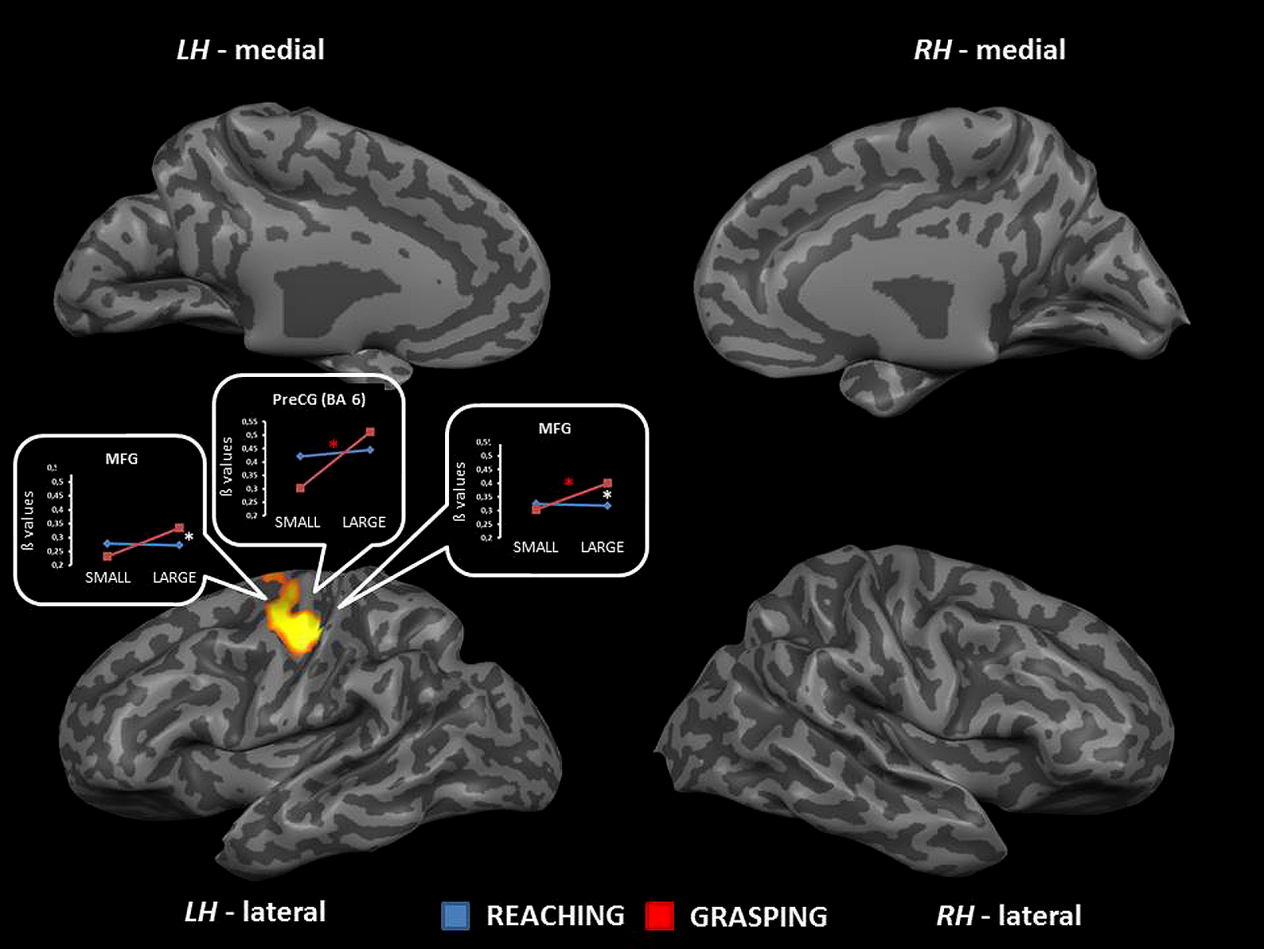
**Brain regions showing significant interaction effects between type of movement and stimulus dimension in TB 7.** The *p* value is set to 0.001, uncorrected for multiple comparisons, cluster size *k* ≥ 13. White asterisks indicate significant effects for the contrast GS > RS; red asterisks indicate significant effects for the contrast GS > RS. (PreCG, precentral gyrus; MFG, middle frontal gyrus; BA, Brodmann area; LH, left hemisphere; RH, right hemisphere; medial, medial view; lateral, lateral view).

The interaction between type of movement and stimulus dimension did reach significance within the left PreCG [BA 4: *F*_(1,68)_ = 22.98; and 6: *F*_(1,68)_ = 19.30] and the left MFG [BA 6; *F*_(1,68)_ = 18.78]. Inspection of the interaction indicated that RS and RL were associated with a similar level of activation, while GL showed a signal level which was higher than that observed in GS. The contrast GL > GS was significant in both regions of the PreCG, while the comparison GL > RL underlined significant differences within the PreCG (BA 4) and the MFG. (see **Table 2** and **Figure 5**). No further significant effects were observed.

### TB 8

**FIGURE 6:**
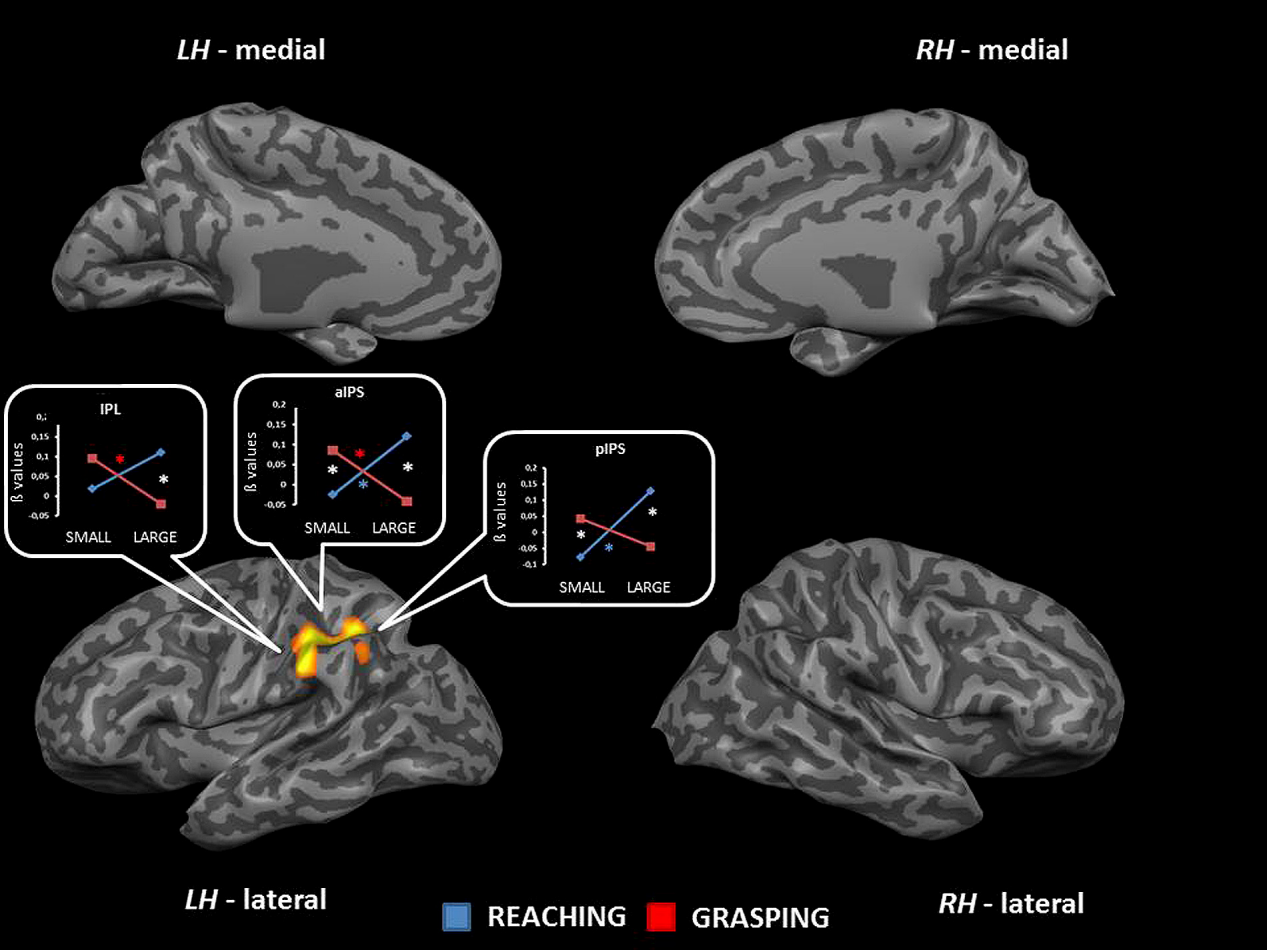
**Brain regions showing significant interaction effects between type of movement and stimulus dimension in TB 8.** The *p* value is set to 0.001, uncorrected for multiple comparisons, cluster size *k* ≥ 13. White asterisks indicate significant effects for the contrast GS > RS and RL > GL; red asterisks indicate significant effects for the contrast GS > RS; blue asterisks indicate significant effects for the contrast RL > GL. (IPL, inferior parietal lobule; aIPS, anterior intraparietal sulcus; LH, left hemisphere; RH, right hemisphere; medial, medial view; lateral, lateral view).

The interaction between type of movement and stimulus dimension was significant within three different sectors of the IPL corresponding to the lateral surface of the left IPL [*F*_(1,68)_ = 31.13], the left aIPS [*F*_(1,68)_ = 28.91], and the left pIPS [*F*_(1,68)_ = 23.80]. Inspection of the interaction patterns revealed a similar pattern of results for all regions, that is RL was associated with a higher level of activity than RS, and the level of activity for GS was higher than that found for GL. The contrast RL > RS was significant within both aIPS and pIPS, while the comparisons GS > GL underlined significant effects within IPL and aIPS. In addition, the contrasts GS > RS and RL > GL were significant in both sectors of the IPS (aIPS and pIPS – see **Table 2** and **Figure 6**). No further significant effects were observed.

### TB 9 and 10

Random effect analysis performed on TBs 9 and 10 did not bring any significant result.

## DISCUSSION

Neuroimaging investigations on grasping in humans have revealed similarities between human and non-human primates (Grefkes and Fink, 2005). Both domains agree on the idea that both reaching and grasping, even if belonging to the same act, are supported by the recruitment of different brain regions. A more dorsomedial network, involving the SPOC, the mIP and the dorsal premotor cortex would mainly subserve the reaching component, while a more dorsolateral circuit, including the anterior intraparietal area (AIP) and the ventral premotor cortex would support visuomotor transformation and grip formation. However, this scenario has been put into question by some recent findings (Grol et al., 2007; Cavina-Pratesi et al., 2010; Fabbri et al., 2014) suggesting that both components could be supported by the same circuit, and that the distinction could take place more in temporal rather in qualitative terms. In other words, a common network would supply coding for both aspects, but at different time points. Our results seem to add further support to this view by demonstrating that several key areas belonging to the fronto-parietal network can play a different role according to the stage of the action.

### ANTERIOR INTRAPARIETAL AREA

Intraparietal area is considered the human homolog of monkey aIPS, a brain region involved in visuomotor transformation: this view has been supported by many neuroimaging findings (Frey et al., 2005; Shmuelof and Zohary, 2005; Begliomini et al., 2007a; Stark and Zohary, 2008; Filimon, 2010). The present findings confirm the role played by this area during the visuomotor processes underlying reach-to-grasp movement, but importantly they outline that the kind of computations ascribed to AIP varies as time unfolds. In fact, AIP seems to code for type of movement in TB 4 and 6, and for stimulus dimension in TB 8. TB 4 and 6 refer to 4 and 6 s after *stimulus* onset, and 2 and 4 s after *movement* onset, respectively. Therefore the observed effects might refer to planning rather execution processes, since we know that the maximum peak of the hemodynamic response is reached around 6 s. Differently, TB 8 (that is 8 s before *stimulus* onset and 6 s after *movement* onset) refers to a time point at which the hemodynamic response mainly reflects brain activity related to the execution rather than the planning phase. This scenario invites to make several considerations. Firstly, AIP begins to differentiate between movements rather early. Even if the hemodynamic response around the 4th second is still far from reaching its maximum, AIP already discriminates among conditions with different accuracy requirements. Along these lines, a recent evoked related potentials (ERPs) reach-to-grasp study showed that processing occurring in AIP starts at the very early stages of action planning, when the translation of object representation into a motor program occurs (Bozzacchi et al., 2012; Verhagen et al., 2013; see also Tarantino et al., 2014). Secondly, during the planning and the execution stages, the role played by AIP seems to change: at the very beginning of action planning (TB4), AIP activity seems to be devoted to computations related to accuracy as witnessed by the fact that AIP activity is significantly higher for the GS than for the RS condition. In a later stage (TB6) RL activity is significantly stronger with respect to the GL condition; finally, during execution (TB8) effects observed in the left parietal region seem to suggest that AIP is coding for both accuracy in grip formation and spatial computing, necessary to approach the object with the right trajectory. A recent study on macaques (Lehmann and Scherberger, 2013) has indeed demonstrated that AIP contains different neuronal populations dedicated to either grip formation or spatial encoding. While neurons devoted to grip formation appear to be more active during action execution, neurons coding for spatial computing are active during both action planning and execution. Therefore we could expect that during planning AIP activity could reflect spatial processing rather than grip formation. Along this line, RL might require “more” spatial computing than GL, since the hand cannot count on palm and fingers to reach the goal, but just on hand knuckles. Therefore in this condition the spatial analysis necessary to support RL might require additional resources, as shown by the RL > GL effect in both planning and execution stages. Why we observe this effect for the large but not the small object might be due to the fact that GL and RL are physically distinct movement (GL involves palm and fingers, RL only the back of the hand). In comparison, GS and RS are more “similar” from a spatial point of view (GS involves only two fingers). We are aware that this hypothesis stems from neurophysiological data and would need further investigations in humans to be fully confirmed. However, at TB 8, that is during action execution, both GS and RL appear to be associated with significantly higher levels of activity than those noticed for RS and GL, respectively. Thirdly, stimulus dimension seems to play a significant role only at later stages, corresponding to action execution: the small stimulus seems to be associated with significantly stronger activity with respect to the large stimulus, but only for reach-to-grasp movements. This may suggest that during action execution AIP might be chiefly devoted to matters concerned with accuracy requirements related to the on-line control of a sophisticated grasping movement like GS. It is known that prehension of objects with small surfaces (relative to finger size) requires a larger degree of visual feedback (Bootsma et al., 1994), and that the kinematic profile of the hand is disproportionally altered when grasping small objects without visual guidance (Chieffi and Gentilucci, 1993). Berthier et al. (1996) also showed that as visual information and object size decreased, subjects had longer movement times, slower speeds, and more asymmetrical hand-speed profiles. In line with previous evidence we suggest that, during the prehension of small objects, AIP activity could increase in order to transform object-centered target representations into motor space on the basis of incoming visual information of the moving arm (Grol et al., 2007). The emphasis here is on control, as the modulatory influences of object size on the dorsolateral circuit are related to the execution phase of the prehension movement.

Differently, during the execution of reaching movements AIP activity was higher for movements performed toward the large than the small object. This is a puzzling result given that evidence in humans indicates that the kinematical organization of reaching is affected by the precision requirements related to intrinsic features of objects such as size (MacKenzie et al., 1987; Gentilucci et al., 1991; Castiello, 2001). In this perspective we would have expected increased AIP activity as a reflection of the need for more on-line control required by reaching small objects. Although we do not have a firm explanation regarding this specific aspect of our results, it is worth clarifying that previous experiments in humans have employed a variety of tasks to investigate the neural correlates of reaching. They include reach-to-touch (Levy et al., 2007; Cavina-Pratesi et al., 2010), pointing (DeSouza et al., 2000; Astafiev et al., 2003; Connolly et al., 2003; Fernandez-Ruiz et al., 2007; Hagler et al., 2007), and joystick manipulation (Grefkes et al., 2004). These tasks differ widely in the extent of arm movement, purpose, and cortical recruitment (Culham and Valyear, 2006; Culham et al., 2006; Filimon et al., 2009). Therefore, we cannot exclude that adopting a different task might have brought to different outcomes.

Another aspect of the present findings worth mentioning is that in TBs 4 and 6 AIP involvement is bilateral. This might be due to a bidirectional crosstalk between the two homologous areas or, more simply, to the fact that in TB 4 and 6 the action has yet to be executed, participants could theoretically grasp or reach the object with either the left or the right hand (Binkofski et al., 1999; James et al., 2003; Culham and Valyear, 2006; Culham et al., 2006). The need for bilateral AIP contribution for hand shaping has been demonstrated by some previous findings (Culham et al., 2003; Ehrsson et al., 2003).

### CINGULATE CORTICES

Cingulate cortices (aCC and mCC) are known to play a fundamental role in decision making processes. This aspect is of particular interest since each object we interact with can be grasped in several ways (Fagg and Arbib, 1998; Rizzolatti and Luppino, 2001). The chosen grip depends on object visual properties, but also on object meaning and on what the agent wants to do with the object. In this perspective, the selection of one amongst the possible ways of grasping an object does not only rely on the visual intrinsic properties of the object, but also on action goals (Cohen and Rosenbaum, 2004; Ansuini et al., 2007, 2008). Therefore decisions regarding which motor program has to be implemented should occur before movement execution, that is during action planning. Accordingly, here we found that aCC (bilaterally) and mCC (left hemisphere) show a significant interaction effect between type of movement and stimulus dimension during movement planning (TBs 4, 5, and 6). To elaborate, in TB 4 the aCC distinguishes among movements performed toward the small object, with higher levels of activity for GS than for RS. In TB 5 such difference persists, but also stimulus dimension appears to play a role for grasping movements. The level of activity for GS was significantly different from GL. At TB 6, the mCC shows higher levels of activity for RL rather than GL. These results agree with previous evidence indicating the aCC and mCC are regions involved in action selection (Lau et al., 2004; Cavina-Pratesi et al., 2010; Rowe et al., 2010). According to these studies, the aCC and mCC play a fundamental role in the selection among competing responses (movement schemata, in this case) together with other areas of the fronto-parietal network. In addition, as for AIP, activity within the CC seems to change during different stages of action planning: at a very early stage (TB 4 and 5) aCC seems to be responsible for choosing the most appropriate motor program on a more accuracy-based criteria: we know from previous studies that GS is usually associated with stronger activity in visuomotor related areas as well as longer reaction time suggestive of a more demanding planning phase. In TB 6 the mCC seems to be more engaged for the coding of type of movement as far as the large object is concerned (RL > GL). Although this result can sound a bit counterintuitive since the general agreement considers grasping more “demanding” than reaching, it is also known that the mCC is involved in the integration between the effector and the target during reaching planning (Beurze et al., 2007): the fact that the stronger activity is associated with the large object can be due to the larger amount of visuospatial information processing necessary when the target of the action is a large object (Tarantino et al., 2014).

Overall, the results concerned with CC activity seems to indicate that a more anterior sector of this regions is engaged in the processing of “accuracy” at the very early stages of action planning (TB 4 and 5), whereas a more posterior sector (mCC) seems to be more devoted at a later stage (TB 6) to spatial coding and the matching between effector and target.

### MIDDLE FRONTAL GYRUS

Interaction effects became significant in the MFG within the *right* hemisphere at TB 5 and within the *left* hemisphere in TB 7. The MFG belongs to the dorsal sector of the premotor cortices (PMd) and it is known to be involved in motor planning (Davare et al., 2007; Begliomini et al., 2007b, 2008; Fabbri et al., 2014; Tarantino et al., 2014). Interaction effects in TB 5 show greater activity for GS in respect to RS, while no effects are evident for movements performed toward the large object. This finding may reflect the need of higher levels of accuracy required by the planning and the subsequent execution of a precise grasping movement. The fact that only the ipsilateral MFG shows significant effects it is not surprising: several studies have advanced the role of the right PMd in online monitoring of action planning and execution, regardless of the side of the effector (Davare et al., 2006; Begliomini et al., 2008). At TB 7, when the action is about to start, it is the left MFG to show interaction effects between type of movement and stimulus dimension. This region of the MFG seems to be sensitive to stimulus dimension while grasping (GL > GS) but not while reaching objects. This pattern of results becomes significant when the target of the action is the large object, with higher levels of activity for GL with respect to RL. This seems to indicate that, while switching from planning to execution, the left MFG is significantly more alerted for the GL condition. Previous results indicate that the MFG, together with aCC, could represent a part of the neural circuit supporting the selection for action (Lau et al., 2004). The fact that GL is associated with the strongest level of activity seems to suggest that grasping a large object might require additional control since all the fingers have to act in concert to achieve a hand posture suitable for grasping the large object.

A particular worth mentioning is the discrepancy in anatomical coordinates of the MFG in the right and the left hemisphere. More precisely, the MFG region showing significant effects in the right hemisphere (TB 5) appears to be more ventrally and anteriorly located in respect to the MFG regions showing significant effects in the left hemisphere (TB 7). However, when movements with the right hand are performed, MFG activity typically reflecting on-line monitoring is usually detected in regions more anteriorly located in respect to their homologous in the left hemisphere. However, the right MFG shows significant effects at TB 5 (action planning) while the left MFG appears to be significantly engaged only at TB 7 (action execution). The different stages of the action and the consequent different contribution of MFG to the ongoing process, together with the laterality of the effector used to perform the action might explain this anatomical discrepancy. Further studies are needed to confirm this result, especially in light of some very recent neurophysiological findings investigating the role of premotor cortices during the execution of a specific task and the refraining from performing it (Bonini et al., 2014). The study indicates that MFG seems to be involved in both situations, suggesting that this region encodes action representations also when the actions is not performed or delayed, which is actually the case of our paradigm (remember the 2 s delay before action initiation).

### PRECENTRAL GYRUS

The PreCG hosts the primary motor cortex, the brain region controlling the execution of proximal and distal motor acts of our body. Here, we find significant interaction results within the left PreCG at TB 7, that is during motor execution. The pattern of activity within this region indicates that while reaching small or large objects does not lead to any difference, the act of grasping a large object leads to significant increases with respect to both reaching for the large object and grasping the small object. Similar findings have been reported in several previous studies (Begliomini et al., 2007a,b) and it is suggestive of a need for additional motor control to coordinate palm and fingers: in fact GL is the only condition in which fingers and palm have to be perfectly coordinated in order to acquire the right configuration as to hold the object.

## CONCLUSION

We examined interaction effects between grasping and reaching movements performed toward small and large spherical objects within areas belonging to both the “reaching” and the “grasping” circuit. We observed that similar areas seem to be sensitive to both types of movements, providing further confirmation to the idea that the neural underpinnings of reaching and grasping may overlap in both spatial and temporal terms (Verhagen et al., 2013). However, from the results, it also emerges the possibility that, although responsive for both actions, they show a significant predominance for either one of the two actions and such a preference is evident on a temporal scale. Further studies are needed to better disentangle the temporal dynamics of medial and lateral pathways interactions, exploring patterns of functional and effective connectivity among these regions.

## Conflict of Interest Statement

The authors declare that the research was conducted in the absence of any commercial or financial relationships that could be construed as a potential conflict of interest.

## Footnotes

1 http://www.ab-acus.com/products.html

2 http://www.fil.ion.ucl.ac.uk/spm/ext/#ArtRepair

3 www.ansir.wfubmc.edu
